# Recent Progress in Porous Fused Aromatic Networks and Their Applications

**DOI:** 10.1002/smsc.202000007

**Published:** 2020-10-21

**Authors:** Ishfaq Ahmad, Javeed Mahmood, Jong-Beom Baek

**Affiliations:** ^1^ School of Energy and Chemical Engineering/Center for Dimension-Controllable Organic Frameworks Ulsan National Institute of Science and Technology (UNIST) UNIST 50 Ulsan 44919 Republic of Korea

**Keywords:** conjugated microporous polymers, covalent organic frameworks, fused aromatic networks, network polymers, porous polymers

## Abstract

Porous materials are ubiquitous in nature and play central roles in ecosystems and human life. Porous fused aromatic networks (P‐FANs) are emerging as a new class of porous organic materials, which can be precisely constructed from organic precursors via the formation of irreversible fused aromatic rings. Despite the luculent advantages offered by P‐FANs, the research community has conventionally focused on more synthetically accessible covalent organic frameworks (COFs), which mostly involve reversible condensation reactions. An overview of the trend in the field of P‐FANs and their possible applications in different fields, including proton conduction, catalysis, gas storage, supercapacitors, and optoelectronics, is presented herein.

## Graphical abstract

Porous materials are present everywhere in nature and play vital roles in ecosystems and human life. Fused aromatic networks (FANs) are an emerging class of porous organic materials. Their versatile covalent linkages, dimensionality, pore controllability, and conductivity make FANs broadly interesting and highly promising materials for energy‐conversion and storage devices.

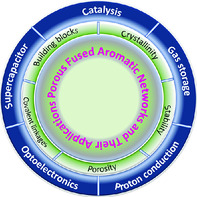

## 1 Introduction

A porous structure is a substance having a solid matrix with interwoven voids. Since ancient times, porous materials have been used for different purposes, and countless porous materials exist in nature.^[^ ^1^ ^]^ These porous materials can be classified as synthetic/natural,^[^ ^2^ ^]^ inorganic,^[^ ^3^ ^]^ or organic structures based on their structural compositions.^[^ ^4^ ^]^ Examples of natural porous materials include sandstone, honeycomb, wood, bread, foam, volcanic rocks, human lungs, and many more. To imitate these natural porous features, material scientists and chemists have attempted to develop artificially porous materials.^[^ ^5^ ^]^ A diverse number of nanoporous organic polymers can be synthesized by connecting different organic building blocks using covalent bonds. Examples include covalent triazine frameworks (CTFs),^[^ ^6^ ^]^ fused aromatic networks (FANs),^[^ ^7^ ^]^ conjugated microporous polymers (CMPs),^[^ ^8^ ^]^ covalent organic frameworks (COFs),^[^ ^4^ ^]^ polymers of intrinsic microporosity (PIMs),[^8^, ^9^] hyper cross‐linked polymers (HCPs),^[^ ^10^ ^]^ porous organic cages,^[^ ^11^ ^]^ and extrinsic porous molecules.^[^ ^12^ ^]^ All of the above polymers have large specific surface areas and tunable pore dimensions. Among them, porous fused aromatic networks (P‐FANs) are a distinct class of porous organic materials. Reported by Jiang and coworkers for the first time in 2011, P‐FANs comprise irreversible fused aromatic linkages.^[^ ^13^ ^]^ This aspect distinguishes them from other types of COFs, such as boroxine, imine, imide, triazine or hydrazine‐based COFs. Materials with irreversible fused aromatic linkages are crucial for developing porous organic materials and possess many advantages, including low density, porosity, good thermal and chemical stability,^[^ ^14^ ^]^ controllable pore dimensions, and designable functions.

The numbers and dimensional ordering of the reacting groups within the building blocks govern the formation of the framework skeleton in two (2D) or three (3D) dimensions.^[^ ^15^ ^]^ In 2D P‐FANs, precursors are covalently linked with fused aromatic linkages into 2D atomic layers that expand further into a planar structure via interlayer *π*–*π* interactions. In 3D P‐FANs, the network structure is preserved by the geometry of the building units.^[^ ^16^ ^]^ P‐FANs have been widely studied from a chemistry perspective because of their environmental stability, tunable skeleton, and controllable pore size.^[^ ^17^ ^]^ From a physics perspective, the extended *π*‐conjugated network offers continuous delocalization of electrons along with the framework, which allows the migration of electrons, excitons, holes, and molecules.[^17^, ^18^] Potential applications, including catalysis,^[^ ^19^ ^]^ proton conduction,^[^ ^20^ ^]^ gas storage,[^10^, ^21^] semiconductors,[^17^, ^22^] and energy conversion and storage,^[^ ^13^ ^]^ have been the leading research foci in the field of P‐FANs over the past few years.

## 2 Structural Design, Synthesis, and Advantages of P‐FAN Structures

The diagram of topology design focuses on illustrating the direction of covalent bond formation and guiding the polygonal backbone growth. Monomers (building blocks) having rigid backbones, in which the functional groups are correctly oriented, are highly desired to dictate the direction of covalent bond formation. The resulting covalent bonds steer the structural direction and regulate the location of the next reacting group. This order for every connection determines the direction of chain growth to construct polygonal structures. The topology design blueprint and different geometry matching of precursors lead to the formation of the ordered structure.^[^ ^23^ ^]^ The structures and pores of P‐FANs depend on the rational selection of matching building blocks, which in turn guide the development of the polygonal architecture. The polygons consist of fused aromatic cores and linkers. Their geometry determines the polygonal architecture and dimensions, which further direct pore size and shape.^[^ ^16^ ^]^

**Figure 1:**
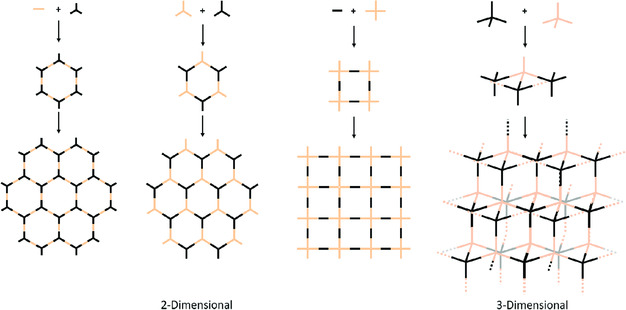
Topological diagrams showing possible combinations of building units with different geometries that can be used to construct P‐FANs.

With the recent advances in synthesis and computational techniques, the use of molecular design has become customary, especially for the targeted synthesis of more complex skeletons, and has subsequently led to tailor‐made materials. Generally, two parameters dictate both the topology and structure of the predesigned architecture: 1) the type and dimensional distribution of the functional moieties in the building blocks and 2) the corresponding reactions that bind the precursors.^[^ ^24^ ^]^ Different geometric matches of the precursors lead to the formation of distinct polygon skeletons. For example, a hexagonal P‐FAN can be constructed using a combination of a *C*_2_‐symmetric linker with *C*_3_‐symmetric knots,^[^ ^4^, ^25^ ^]^ and connecting *C*_3_ + *C*_3_ topology results in a new type of hexagonal P‐FAN in which both units are alternatively linked together.[^17^, ^26^] Similarly, the combination of *C*_4_ + *C*_2_ topology results in a tetragonal skeleton, in which both the reacting units are alternate groups, which build the network structure (**Figure** **1**).^[^ ^27^ ^]^ This flexibility in controlled structural design has allowed the P‐FAN field to develop a variety of applications.

**Figure 2:**
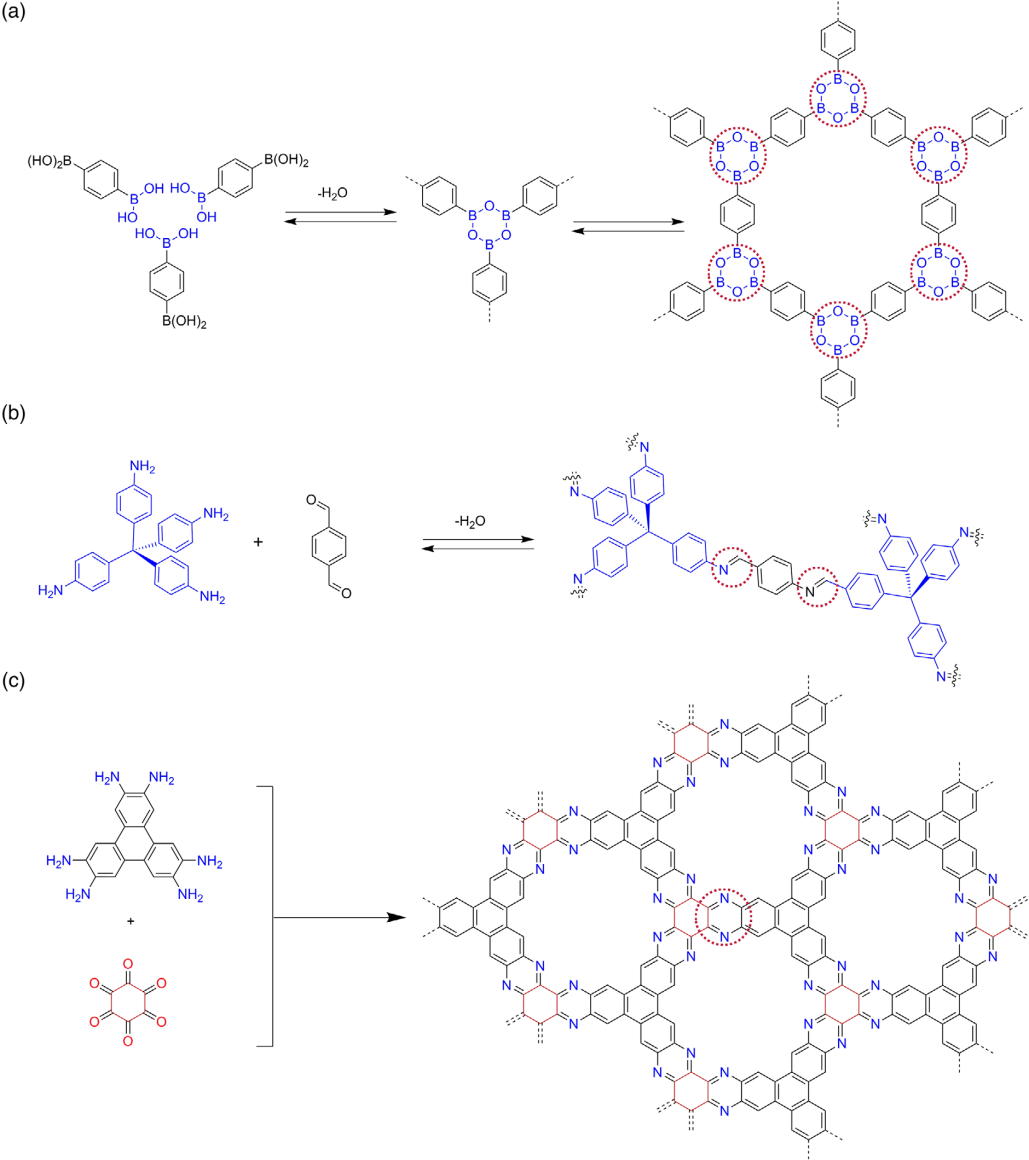
Schematic illustration of representative linkages for constructing structures: a) frameworks containing boroxine linkage, b) imine linkage, and c) phenazine linkage. Reproduced with permission.^[^ ^26^ ^]^ Copyright 2019, Elsevier.

In P‐FANs comprising a polygonal skeleton, organic units are covalently linked to form a fully *π*‐conjugated network structure. P‐FAN synthesis originates from the previous development in other classes of framework materials, especially advances in COF synthesis. The main feature that distinguishes P‐FANs from COFs is the connecting linkage. The formation of P‐FANs involves an irreversible double‐condensation reaction, resulting in fused aromatic rings that impart thermal and physicochemical stability. In contrast, COF formation mostly proceeds through reversible reactions, which provide error correction by self‐healing with the sacrifice of physicochemical stability (**Figure** **2**).^[^ ^16^ ^]^

### 2.1 Synthesis Route for FANs

Despite the availability of countless building blocks, the fabrication of FANs with high crystallinity and porosity is still a complicated task. It is because finding suitable reaction conditions for the formation of thermodynamically controlled products is essential. In other words, to obtain a crystalline product, the synthesis route plays a key role. To reach thermodynamic equilibrium between crystallinity and network forming reactions, finding optimum reaction conditions, including appropriate solvent, temperature, and pressure, is vital. As the synthesis of FANs is a newly established field, three major synthesis routes, such as ionothermal synthesis, solution synthesis, and solvothermal synthesis, are reported.^[^ ^28^ ^]^

### 2.2 Ionothermal Synthesis

Thomas et al. realized the first COF by the trimerization of aromatic nitriles in the presence of zinc chloride as a catalyst via ionothermal synthesis. Later, Jiang and coworkers reported the first ionothermal synthesis of FAN using aluminum chloride as a catalyst. Tetra aminobenzene and triquinoyl hydrate and aluminum chloride were taken in an ampoule. Then it was degassed by vacuum, sealed, and the reaction was conducted at the required condition for overnight.^[^ ^13^ ^]^ Although the FANs synthesized by the ionothermal method showed useful application in a supercapacitor, the crystallinity was still not high enough. To further improve crystallinity, researchers used some other synthesis procedures such as solvothermal and solution synthesis.

### 2.3 Solvothermal Synthesis

A literature survey reveals that a variety of FANs were obtained via solvothermal synthesis. The solvothermal synthesis involves mixing precursors in an ampule with the desired combination of solvent mixtures, followed by a freeze–thaw cycle. After sealing the ampule, the reaction mixture is heated to a designated temperature for a specific time (3–7 days). The next step is the collection of the final FAN product after complete washing. Jiang's group synthesized the first FAN by a solvothermal synthesis method. The FAN was synthesized using a double‐condensation reaction between triphenylene–hexamine (TPA) and *t*‐butyl tetraketopyrene (TP) as precursors. The selection of a proper solvent is vital. It is because the solvent (or solvent mixture combination) not only determines the solubility of reactants and products, but also controls the reaction rate and crystal growth rate. Besides proper solvent combination, the availability of water, which is being generated by the condensation of reactants in the sealed environment, is also necessary to efficiently balance the reaction rate and crystal growth rate.^[^ ^28^ ^]^

### 2.4 Solution/Wet Synthesis

Solution synthesis involves chemical reactions in the solution phase using appropriate solvents to dissolve precursors at proper reaction conditions. Each solution synthesis varies from the others. Some reactions may occur at mild conditions, whereas others require harsh conditions to drive the network forming reaction. Some of the FANs were obtained by the solution synthesis method. Mahmood and coworkers documented the solution synthesis of a 2D FAN (C_2_N structure) using hexaminobenzene (HAB) hydrochloride and ketocyclohexane (HKH) as precursors in trifluoromethanesulfonic acid (TFMSA) as a solvent.^[^ ^26^ ^]^ Solution synthesis possesses certain advantages over solvothermal synthesis, including lower energy usage, mild reaction conditions, distinct reaction selectivity, and many more.

### 2.5 Low Density

P‐FANs are entirely constructed from lightweight elements such as C, N, O, and H, held together by covalent bonds. This results in low mass density and enables high‐performance energy storage and guest molecule uptake.^[^ ^29^ ^]^

### 2.6 Stability

P‐FANs, comprising fully fused aromatic rings linked together via robust covalent bonds, exhibit enhanced physicochemical stability against acids, bases, moisture, and heat compared with most conventional COFs. The versatility of P‐FANs originates with their physicochemical durability, as a result of the presence of fully fused aromatic ring systems. The robust stability expands the adaptability of FANs for further applications.^[^ ^30^ ^]^

### 2.7 Crystallinity

As noted, unlike traditional COFs, which are obtained by thermodynamically controlled reactions with error‐mending characteristics, the synthesis of P‐FANs involves energetically controlled reactions, which result in irreversible covalent bonds.^[^ ^31^ ^]^ This hampers obtaining highly crystalline structures. Improving crystallinity is a vital issue in the further development of P‐FANs. Various methods have been explored to improve crystallinity, including incorporating complementary *π*‐interactions at the periphery between the layers, which substantially enhances the interlayer interactions, and crystallinity.^[^ ^16^ ^]^

### 2.8 Porosity

The primary concern when fabricating P‐FANs and other porous materials is the preservation of porosity.^[^ ^32^ ^]^ From this perspective, multiple design strategies, such as templating methods adopted for other noncovalently bonded porous structures, can also be used for P‐FAN synthesis.^[^ ^33^ ^]^ Another approach is to utilize robust and rigid building blocks to develop a porous architecture. To date, almost all P‐FANs syntheses involve the latter approach, using designed rigorous building blocks to build the prolonged porous architecture.^[^ ^34^ ^]^ The pore size of P‐FANs depends on the length of the building blocks, whereas the topology of the material depends on the shape of building blocks. Building units with aromatic rings is preferable for the efficient construction of porous organic structures.^[^ ^35^ ^]^ The pores of P‐FANs have a regular polygonal shape, which can be designed to be trigonal, hexagonal, tetragonal, rhombic structures with broad domain of sizes from large mesopores to small micropores. The specific surface area of porous materials plays a crucial role in directing some applications, including gas adsorption and storage. The porous architectures of P‐FANs possess a large Brunauer–Emmette–Teller surface area up to 2000 m^2^ g^−1^.^[17b]^

### 2.9 Versatility of Building Blocks and Covalent Linkages

**Figure 3:**
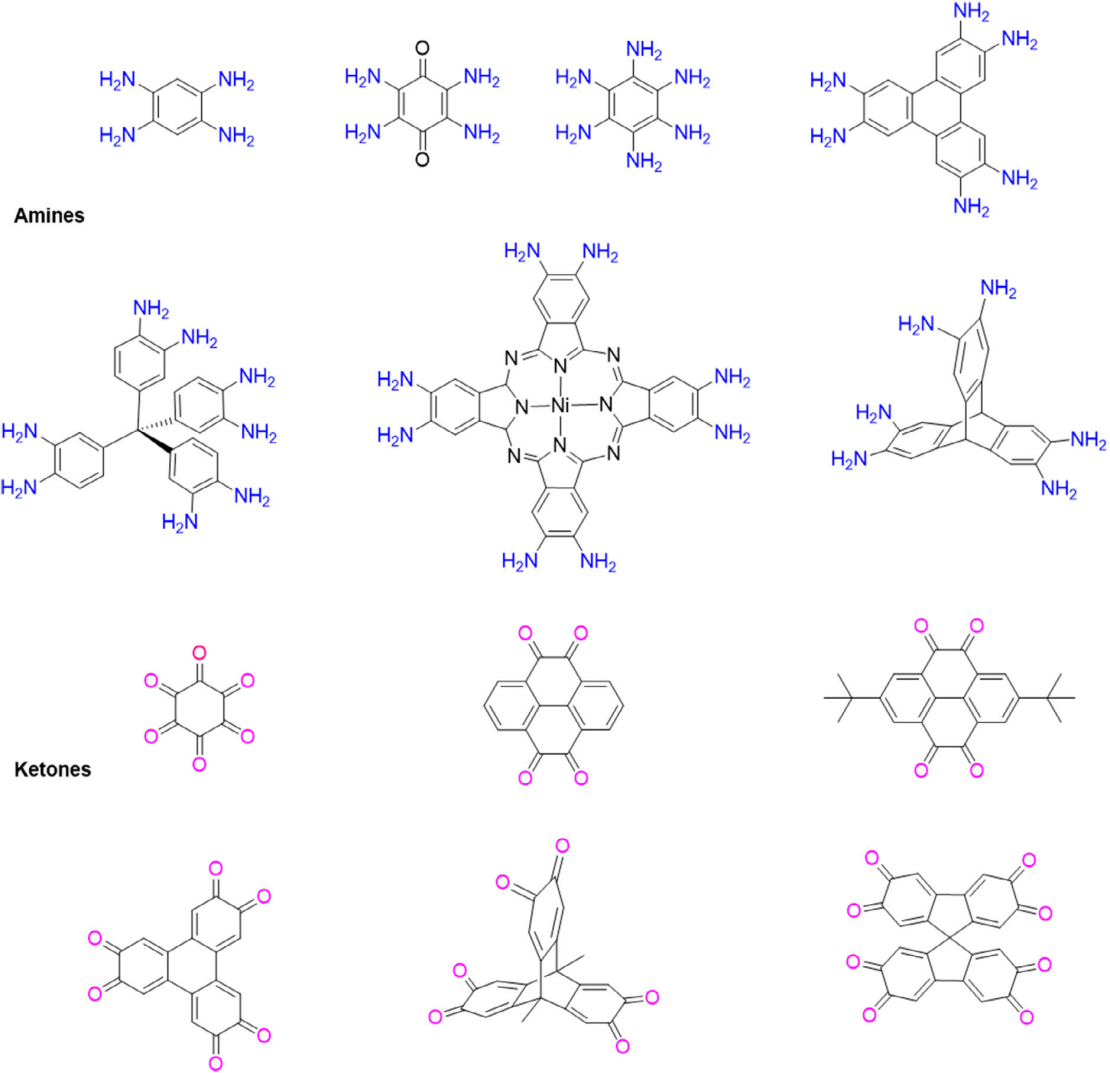
Common building blocks used for the synthesis of P‐FANs.

For the fabrication of P‐FANs, a variety of building units having different sizes and geometry have been practiced. **Figure** **3** shows a summarized list of used functional monomers. Based on the reactive sites, these building units can be divided into ortho‐amines and 1,2‐diketones. The centers of these monomers contain *π*‐conjugated aromatic rings such as triphenylene, pyrene, and porphyrin. The double‐condensation reaction between the diketone group on one precursor and diamine group from the other monomer results in a pyrazine ring as a FAN linker.^[^ ^36^ ^]^ Similarly, from a geometric point of view, these building units can be grouped into 2D and 3D structures based on the directional symmetry of functional groups. The development of complex but ordered structures depends on the structural orientation of these building units. Besides the building block spatial arrangement, the linkage is also crucial for the construction of P‐FANs. The linkages should be reversible or semireversible to assure both crystallinity and self‐error correction. However, some irreversible phenazine bonds have been successfully used for the synthesis of crystalline P‐FANs.^[^ ^27^, ^37^ ^]^ In addition, the phenazine bond can contribute to useful conjugation and conductivity.^[^ ^38^ ^]^ Utilizing these advantages of phenazine linkages, various P‐FANs have been developed and used in many fields.

## 3 Applications of P‐FANs

In this section, we review the potential applications of P‐FANs for proton conduction, catalysis, gas storage, supercapacitors, and electronics.

### 3.1 Proton Conduction

**Figure 4:**
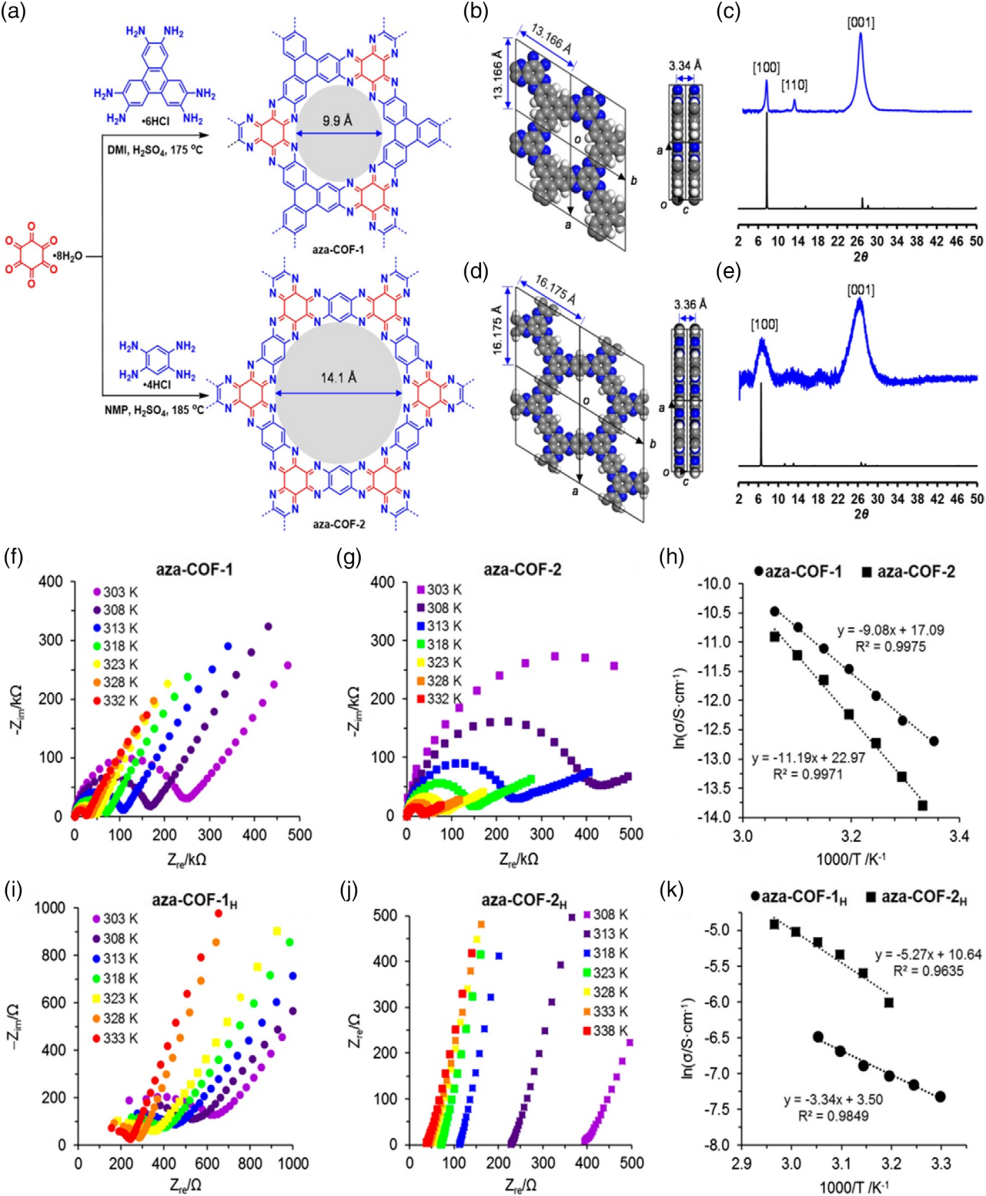
Phenazine‐based frameworks for proton conduction. a) Synthetic illustration of aza‐fused COF. b,d) Graphic view of an optimized structure of aza‐COF‐1 and COF‐2. c,e) simulated (black) and experimental (blue) PXRD of aza‐COF‐1 and COF‐2. f,g) Nyquist plot of aza‐COF‐1 and COF‐2 calculated under a relative humidity (RH) of 97% at variable temperatures, exhibiting the proton‐conduction dependence on temperature. h) At different temperatures, Arrhenius plots of aza‐COF‐1 and COF‐2. i,j,k) Nyquist and Arrhenius plots of acidified samples, aza‐COF‐1_H_ and aza‐COF‐2_H_. Reproduced with permission.^[20d]^ Copyright 2019, American Chemical Society.

As energy consumption increases globally, it is essential to find alternative energy sources, and the hydrogen fuel cell is considered a potential alternative to depleting fossil fuels.^[^ ^5^ ^]^ The synthesis of proton‐conductive materials is key to the fabrication of proton exchange membrane fuel cells.^[^ ^39^ ^]^ Although plenty of microporous polymers have been reported to explore proton conduction,^[^ ^40^ ^]^ the stability of these materials is a significant hurdle.^[^ ^41^ ^]^ One approach to overcome this problem is to construct a stable, fully FAN structure. Recently, Meng et al. reported synthesizing a layered 2D phenazine‐based fused aromatic structure by the condensation reaction of hexketocyclohexane with tetra and hexa‐topic amines (**Figure** **4**).^[20d]^ The built‐in phenanthroline‐like unit promoted the adsorption of water and H_3_PO_4_ to obtain good proton conduction of 10^−3^ and 10^−5^ S cm^−1^ for acidified and pristine analogs, respectively. These architectural approaches promise the further development of steady proton‐conducting materials.

### 3.2 Catalysis

Because of their tunable porosity and substantial specific surface area, porous materials have been used in a wide range of catalysts, including fuel cells.^[^ ^5^ ^]^ Fuel cells are promising energy‐conversion devices because they have the potential ability to convert chemical energy from different fuels directly into electrical energy via electrochemical reactions.^[^ ^42^ ^]^ The oxygen reduction reaction (ORR) is a vital electrode reaction at the cathode of a fuel cell.^[^ ^43^ ^]^ To achieve high energy efficiency, it is necessary to have ORR as close to a reversible condition as possible. To industrialize fuel cells as a green energy‐conversion technology, ORR catalysts have to be stable, economical, and efficient.^[^ ^44^ ^]^ Enormous efforts have been poured into developing stable ORR electrocatalysts for fuel cells. To date, although commercial Pt/C is known to be the best ORR catalyst for fuel cells, its viability is hampered by its scarcity, high cost, poor stability, and methanol tolerance.^[^ ^45^ ^]^

To overcome these obstacles, low‐cost, efficient, and stable electrocatalysts for ORR are in demand. Several effective strategies, including doping or encapsulating metal nanoparticles with carbon‐based materials, have been attempted to increase the efficiency and the electrochemical stability of ORR catalysts.^[^ ^46^ ^]^ However, such catalysts suffer poor stability because pure carbon‐based materials are not able to provide strong interaction with the metal for stable void‐free encapsulation.^[^ ^47^ ^]^ To attain improved interaction, nitrogen‐rich FAN structures, due to their coordinating ability with metals, are gaining attention as a new design for the development of indirect ORR catalysts.^[^ ^26^ ^]^

**Figure 5:**
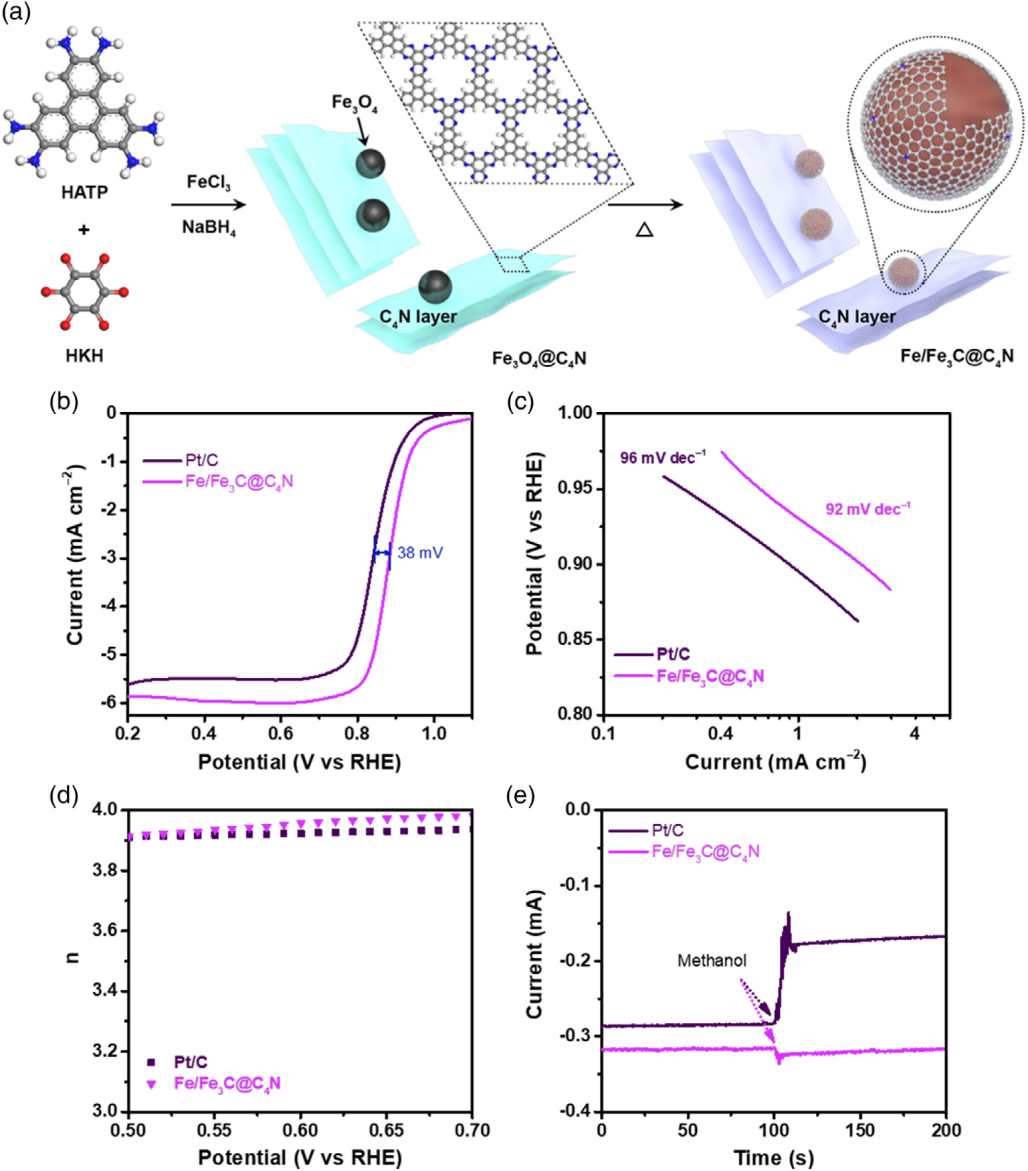
Schematic representation of the design, synthesis, and electrocatalytic performance of the Fe/Fe_3_C@C_4_N catalyst. a) Structural development of the catalyst, exhibiting the formation of Fe_3_O_4_@C_4_N_1_ by reduction with NaBH_4,_ followed by annealing at 800 °C to convert Fe_3_O_4_@C_4_N_1_ to Fe/Fe_3_C@C_4_N. b,c) Linear Sweep voltammetry (LSV) curves and Tafel plots of Fe/Fe_3_C@C_4_N and Pt/C catalysts in 0.1 M aq. KOH solution. d) Electron transfer number of Fe/Fe_3_C@C_4_N and Pt/C catalysts in the potential range of 0.5–0.7 V [vs reversible hydrogen electrode (RHE)]. e) *I–T* curve of Fe/Fe_3_C@C_4_N and Pt/C catalysts in the methanol tolerance test. Reproduced with permission.^[^ ^26^ ^]^ Copyright 2019, Elsevier.

Recently, our group reported an indirect‐contact catalyst comprising iron and iron carbide (Fe/Fe_3_C) nanoparticles sheathed in well‐ordered nitrogenated graphitic networks. The catalyst was synthesized by a double‐condensation reaction between triphenylenehexamine (TPA) and ketocyclohexane (HKH) in the presence of iron chloride, which served not only as a catalyst for the polymerization (aromatization) reaction between TPA and HKH but also as a Fe source for nanoparticle formation. The formation of metal particles involved a chemical reduction with sodium borohydride followed by subsequent pyrolysis at 800 °C for 3 h. This process results in the formation of Fe/Fe_3_C nanoparticles encased in nitrogenated graphitic C_4_N_1_ shells, which were designated Fe/Fe_3_C@C_4_N_1_. The resulting Fe/Fe_3_C@C_4_N catalyst showed a higher catalytic performance and stability compared with that of commercial Pt/C. The Fe/Fe_3_C@C_4_N_1_ catalyst displayed outstanding ORR activity, exhibiting a half‐wave potential of 0.884 V (**Figure** **5**).^[^ ^26^ ^]^

Two other electrocatalysts were also reported from our group, Fe encapsulated in C_2_N layers (Fe@C_2_N)^[19b]^ and a phenazine‐based 2D fused aromatic porous organic network (Aza‐PON).^[19b]^ Both resulting electrocatalysts exhibited ORR performance (with half‐wave potentials of 0.876 V for Fe@C_2_N and 0.826 V for Aza‐PON) better than that of the commercial Pt/C.

The hydrogen evolution reaction (HER) is another crucial electrode reaction for electrochemical water splitting at the cathode, which requires durable, efficient, and cheap catalysts for practical applications. To achieve better efficiency, the HER catalysts must trigger proton reduction as close to overpotential. Tremendous efforts have been devoted to finding highly efficient HER catalysts. Metal nanoparticles for HER electrocatalysts have attracted the interest of the scientific community. Among them, Pt‐based catalysts are still being widely used and are considered the benchmark for HER, due to their fast kinetics and low overpotential in both acidic and alkaline media. However, their high cost and durability issues associated with Pt hinder its wider use. A large variety of nonprecious metal‐based electrocatalysts have recently been documented. However, many of these catalysts typically exhibit acid corrosion, low stability, and higher overpotential issues. Among candidates for efficient catalysts with superior activity and enhanced stability at low costs, ruthenium‐based catalysts are promising alternatives to commercial Pt/C.

**Figure 6:**
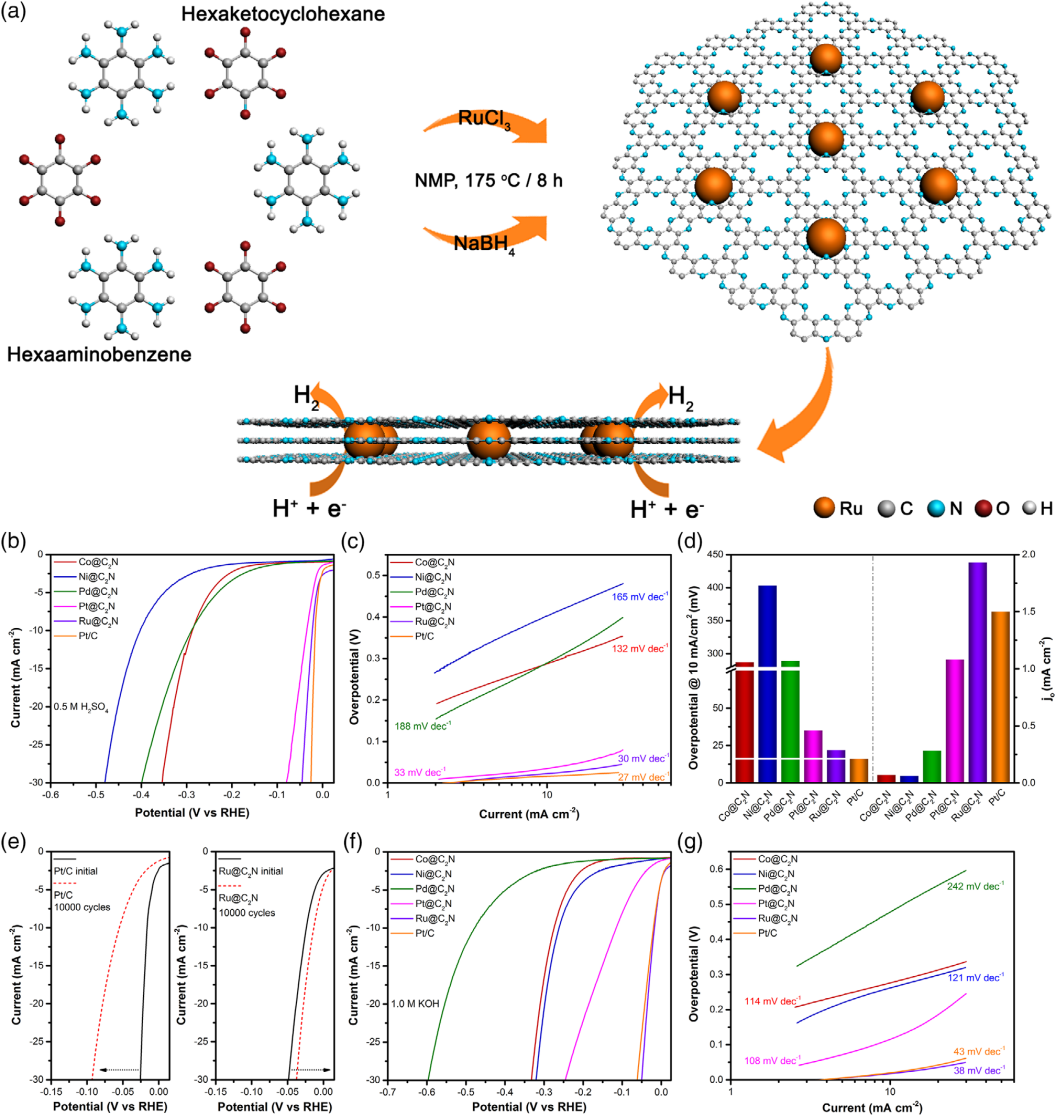
Schematic illustration of the design and synthesis of the Ru@C_2_N catalyst, and its HER activity and stability. a) Synthesis scheme. b) Polarization curves of synthesized electrocatalyst in 0.5 M aq.H_2_SO_4_ solution. c) Tafel slope derived from LSV curves in (a). (d) Overpotentials of the catalysts at 10 mA cm^−2^ (left) and exchange current densities (right). e) Stability test of the Ru@C_2_N and commercial Pt/C. f) Polarization curves of electrocatalysts in alkaline medium (1 M aq. KOH solution). g) Tafel plots are collected from the polarization curve in (e). Reproduced with permission.^[^ ^48^ ^]^ Copyright 2017, Springer Nature.

Mahmood et al. reported a ruthenium‐based electrocatalyst (Ru@C_2_N) that works equally well both in acidic and in alkaline media. The structure and morphology of the Ru@C_2_N catalyst were studied by atomic resolution transmission electron microscopy (AR‐TEM) and powder X‐ray diffraction (PXRD) analysis. TEM images showed that Ru nanoparticles with an average diameter of 1.6 nm were uniformly distributed on the C_2_N network. AR‐TEM revealed that the Ru nanoparticles were highly crystalline, which was also supported by the PXRD results. The Ru@C_2_N catalyst displayed a lower overpotential, delivering a current density of 10 mA cm^−2^ in both acidic (13.5 mV; 0.5 M aq. H_2_SO_4_) and alkaline environment (17 mV; 1 M aq. KOH) (**Figure** **6**). This remarkable performance can be attributed to the semiconductive C_2_N framework, which assists in forming small particles by docking the particles in holes in the matrix.^[^ ^48^ ^]^

### 3.3 Gas Adsorption

Carbon dioxide (CO_2_), methane (CH_4_), and hydrogen (H_2_) are three major gases currently being studied for gas adsorption and storage. CO_2,_ a greenhouse gas, is a significant contributor to global warming.^[^ ^5^ ^]^ Natural gas, consisting of 90% CH_4_, is one of the cheapest sources of global energy and has low energy density. However, because of transportation and storage issues, its utility has not been exploited to its full potential.^[^ ^49^ ^]^ Therefore, sizeable CH_4_ storage capacity at ambient temperature is highly demanded.^[^ ^50^ ^]^ Hydrogen, with high energy content and clean combustion, is ranked as one of the ideal substitutes for a postcarbon future and particularly as an alternative to conventional fossil fuels in automobiles.^[^ ^51^ ^]^

P‐FANs, with their well‐ordered porous structures and high surface areas, seem promising candidates for gas storage.

**Figure 7:**
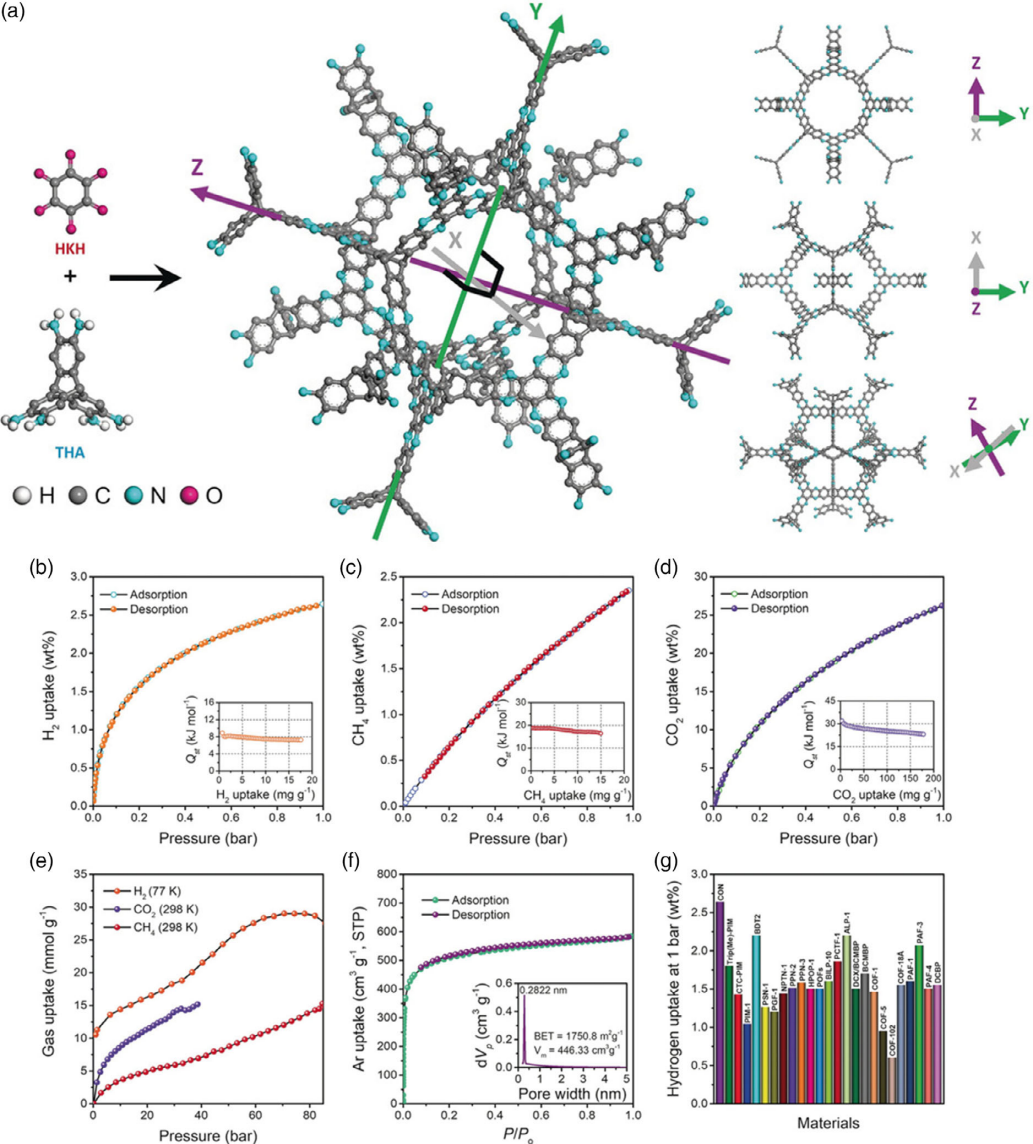
Schematic illustration and gas uptake properties of the 3D‐CON structure. a) Synthesis scheme of 3D‐CON. b) H_2_ adsorption–desorption isotherm at 77 K. Inset: isosteric heat of adsorption (*Q*_st_) for hydrogen measured at two temperatures. c) CH_4_ adsorption–desorption at 273 K. Inset: *Q*_st_ value for methane measured at two temperatures. d) CO_2_ adsorption–desorption isotherm at 273 K. Inset: *Q*_st_ value of carbon dioxide at 273 and 298 K. e) High‐pressure gas uptake (CH_4_, CO_2_, H_2_). f) Argon adsorption‐desorption isotherm measured at 87 K. g) Comparison chart of hydrogen uptake of different organic porous materials. Reproduced with permission.^[^ ^21^ ^]^ Copyright 2018, Wiley.

Mahmood et al. reported a robust 3D cage‐like organic network (CON) structure, realized by the condensation of hexaketocyclohexane (HKH) octahydrate and triptycene‐based hexamine (THA) (**Figure** **7**).^[^ ^21^ ^]^ A monolithic solid gel‐like structure with a dark black color was obtained after completion of the condensation reaction between HKH and THA. After solvent removal and Soxhlet extraction, the material turned bright brown, which indicated the disconnection of aromaticity by barrelene‐like moiety. It is due to the bicyclic core in THA that a durable material with a high surface area is obtained. Taking advantage of its microporous nature, high surface area, and narrow pore size distribution, the 3D‐CON structure was evaluated as a sorbent material for small guest molecules (CH_4_, H_2_, and CO_2_). The low‐pressure H_2_ adsorption–desorption isotherm of 3D‐CON was investigated at 77 K with a pressure ranging up to 1 bar. The hydrogen uptake was found to be 2.64 wt%. To further explore the adsorption behavior of 3D‐CON, a methane adsorption assessment was also conducted in the low‐pressure region. The methane uptake at 1 bar was observed to be 2.40 wt%, which is among one of the best‐recorded values.

Similarly, CO_2_ uptake capacity was also measured at a low pressure, and the obtained uptake value was 26.70 wt%. This CO_2_ uptake of 3D‐CON is the best‐reported value for organic porous materials. The gas sorption studies at a low‐pressure range suggested that the 3D‐CON had still not reached a saturation level at 1 bar. Thus, to assess the high‐pressure gas uptake, sorption analyses for hydrogen, methane, and carbon dioxide were conducted at high pressures as well. The values were found to be 5.8 wt% (H_2_ at 77 K, 85 bar), 24.5 wt% (CH_4_ at 298 K at 80 bar), and 70 wt% (CO_2_ at 298 K and 35 bar). These excellent results indicate the massive potential of P‐FANs as adsorbent materials.

### 3.4 Supercapacitors

**Figure 8:**
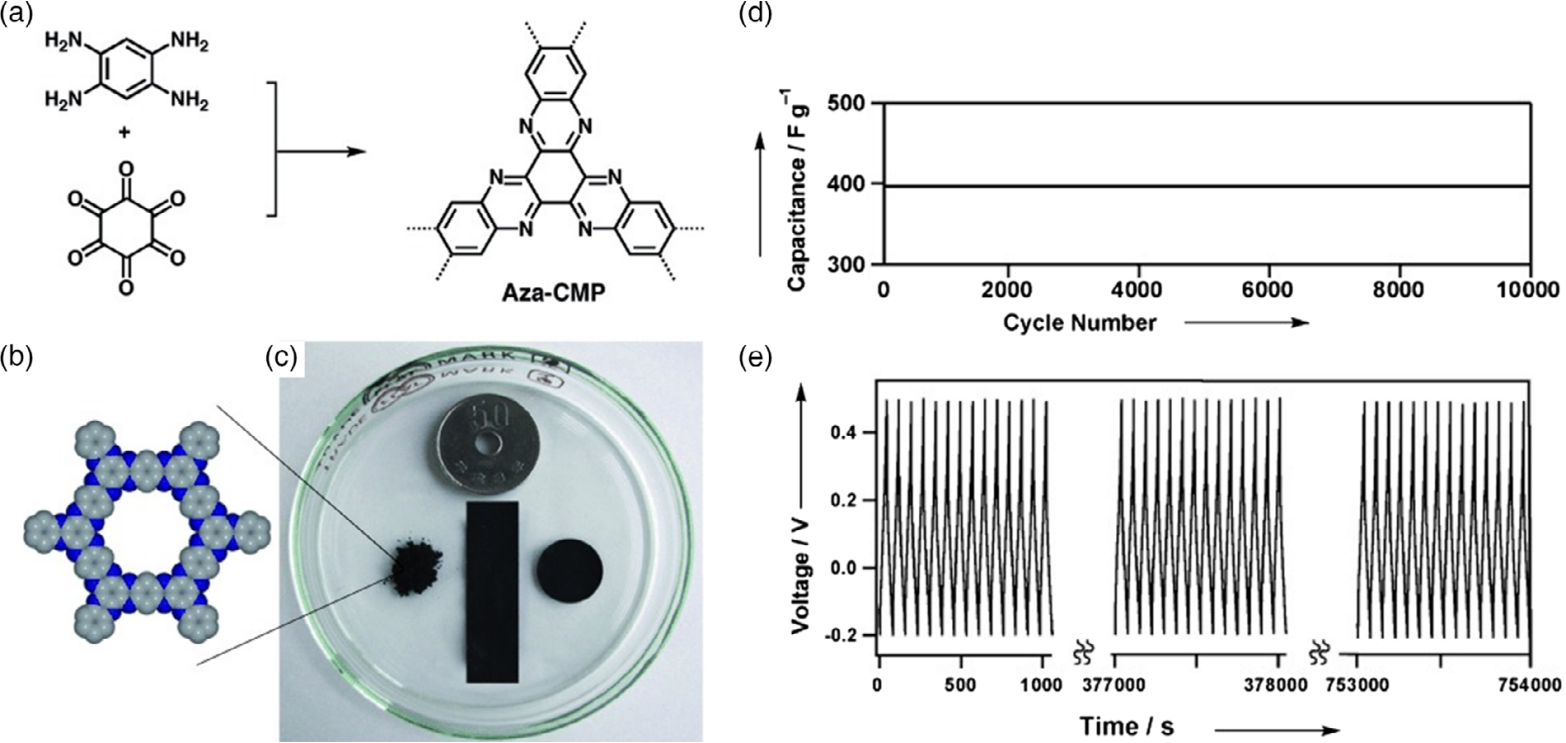
Conjugated FAN for supercapacitor study. a) Synthesis scheme of microporous *π*‐conjugated polymer (Aza‐CMP). b) Graphic view of the pore shape of aza‐CMP. c) Photographs of the obtained powder and thin film of Aza‐CMP. d) Capacitance of Aza‐CMP@350 over 10 000 cycles at a current density of 5 A g^−1^. e) Charge–discharge profile of Aza‐CMP@350 during 10 000 cycle tests. Reproduced with permission.^[^ ^13^ ^]^ Copyright 2011, Wiley.

To meet increasing energy demands, new materials and systems are also required for storing renewable and clean energy with enhanced efficiency. Among them, supercapacitors and rechargeable batteries are booming with expanding applications, as a feasible option to capture energy by reversibly transforming chemical energy into electrical energy.^[^ ^52^ ^]^ Supercapacitors circumvent the drawbacks of rechargeable batteries, like performance snags and low‐power density, with improved performance, low maintenance cost, long life cycle, and high‐power density. In supercapacitors, energy is gathered in electric double layers by accumulating charges at the interface between an electrode and electrolyte. Carbon‐based nanoscale materials, such as carbon nanotubes, graphene, hybrid carbon materials, and aerogels, have been widely investigated to boost the performance of the capacitor. Despite the exhaustive efforts to develop electrode materials, huge capacitance, durability, and high energy density have not yet been achieved and are still the ultimate targets.^[^ ^53^ ^]^ For the first time, P‐FANs were explored for supercapacitive energy storage by Kou et al. The phenazine‐based FANs were ionothermally synthesized by double condensation between hexaketocyclohexane and tetraminobenzene at different temperatures ranging from 300 to 500 °C.^[^ ^13^ ^]^ The phenazine‐based FAN displayed excellent performance and durability without a change in capacitance (397 F g^−1^) at a current density of 5 A g^−1^ even after 1000 cycles (**Figure** **8**). The capacitance reported here is comparable with the best‐known supercapacitor.^[^ ^54^ ^]^ The fully fused aromatic framework containing a phenazine ring and regular microspores facilitated the formation of the electrostatic charge‐separation layer. The outstanding results reported by Kou et al. demonstrate the enormous potential of P‐FANs as energy‐storage devices.^[^ ^13^ ^]^

### 3.5 Optoelectronics

Numerous conducting materials based on organic structures have been designed by fine tuning their bandgap and highest occupied molecular orbitals/lowest unoccupied molecular orbitals levels. Among them, 2D‐COFs have also been used as organic semiconductors for potential applications in photovoltaics,^[^ ^55^ ^]^ optoelectronics,^[^ ^31^ ^]^ and (photo) electrocatalysis.^[^ ^56^ ^]^ COFs were utilized to construct electronic and optoelectronic devices for the first time in 2008. However, because they lack the intrasheet *π*‐conjugation and the chemical stability of a typical COF, electronic devices synthesized from these materials frequently show lower performance than expected.^[^ ^53^ ^]^ To address these limitations, it would be desirable to construct robust, chemically stable yet fully conjugated materials. Because they inherently combine porosity and *π*‐conjugation, P‐FANs have been explored for such applications. The materials also display unique electrical and optical properties when they are logically functionalized with photoelectric moieties. In 2D P‐FANs, the *π*–*π* interaction in the structure induces electronic delocalization among the *π* orbitals in the layers, allowing the transportation of excitons and charge carriers through the network.^[^ ^53^ ^]^

**Figure 9:**
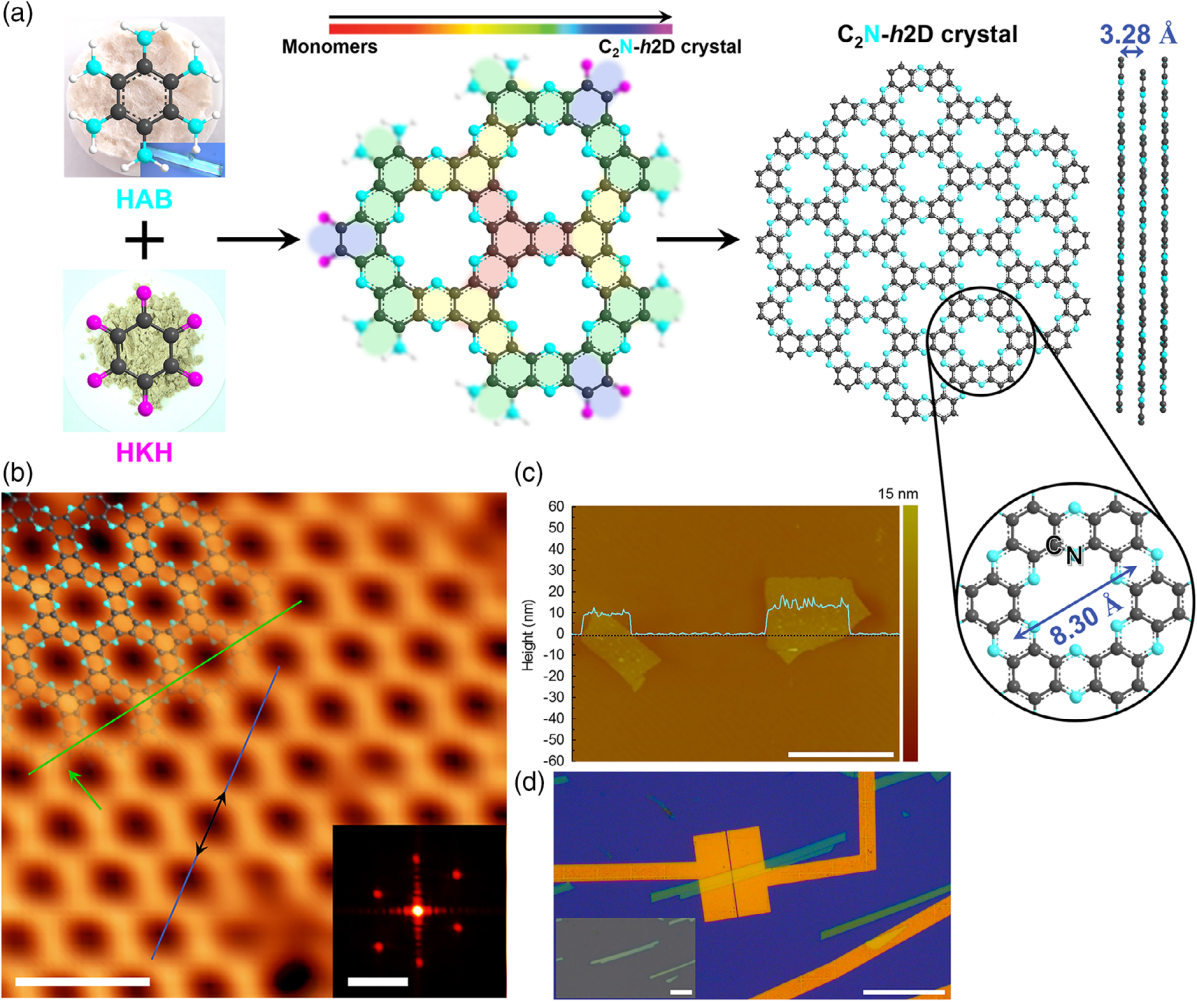
Preparation of C_2_N and STM characterization. a) Schematic illustration of the reaction between HAB and HKH to yield C_2_N. b) Atomic resolution scanning transmission electron microscopy topology image of C_2_N on Cu(111). c) The atomic force microscopy (AFM) image of C_2_N flake with height profile along the cyan–blue line (scale bar is 7 μm). d) Optical microscopy image of C_2_N FET prepared on a SiO_2_ (300 nm)/n^++^ Si wafer. Reproduced with permission.^[17a]^ Copyright 2015, Springer Nature.

Recently Mahmood et al. reported a planar 2D phenazine‐based FAN structure synthesized by an aromatization reaction using hexaminobenzene (HAB) trihydrochloride and hexaketocylohexane (HKH) octahydrate as precursors. The resulting 2D‐layered C_2_N structure contained uniformly allocated nitrogen atoms and periodic holes.^[17a]^ Moreover, the existence of flat bands close to the Fermi level and the extent of the bandgap revealed that C_2_N (where phenyl rings are connected by pyrazine rings) was quite distinct in the electronic structure to other widely studied 2D structures. To explain the electrical properties of C_2_N, field‐effect transistor (FET) devices were fabricated using C_2_N as an active layer, with a thickness of ≈8.0 nm. C_2_N had a direct bandgap of 2 eV and an on/off ratio as high as 10^7^ in the FET devices (**Figure** **9**).

**Figure 10:**
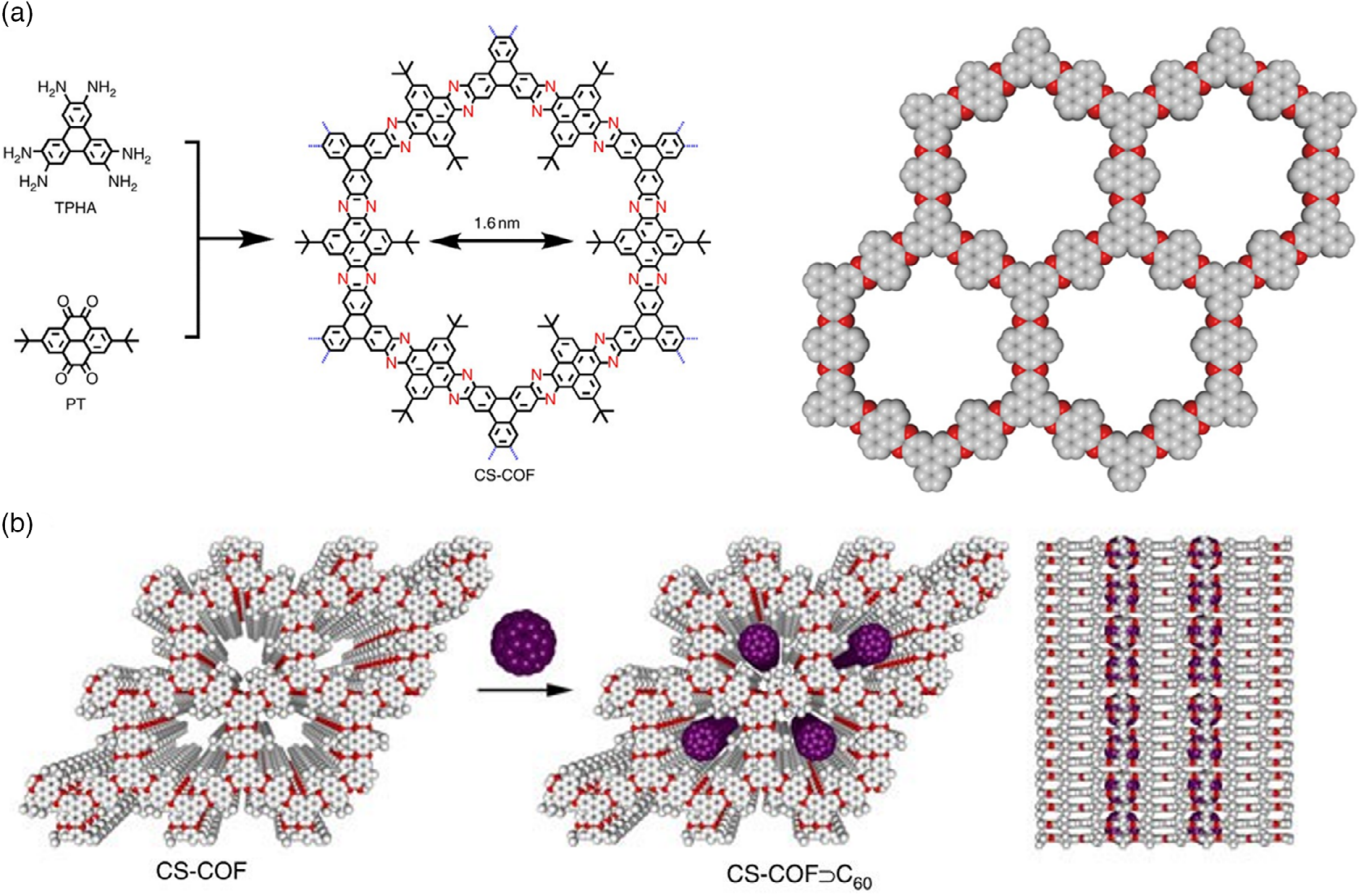
a) Schematic illustration and graphic representation of the CS‐COF skeleton (grey: carbon, red: nitrogen, ter‐butyl group, and hydrogen atoms are excluded for simplicity). b) Schematic representation of the synthesis of CS–COF–C60 by sublimed crystallization of fullerene in the open 1D channels (white: carbon, red: nitrogen; purple: fullerene). Reproduced with permission.^[17c]^ Copyright 2013, Springer Nature.

Guo et al. have also reported a 2D‐layered phenazine‐based FAN, prepared by the condensation reaction between hexamine–triphenylene and tertiary butyl tetraketopyrene as monomers.^[^ ^57^ ^]^ The fully *π*‐electron conjugation spanned the entire layer of CS–COF, which suggests the usefulness of the structure in optoelectronics. The charge carrier mobility of CS–COF was found to be 4.2 cm^2^ V^−1^ s^−1^ using the flash photolysis time‐resolved microwave conductivity method, putting it among the best hole‐transporting organic materials. Moreover, the presence of micropores in the CS–COF structure allows integral functionalization and forms a bicontinuous order donor–acceptor system, which can be created by physically filling the pores of the CS–COF with fullerene molecules (C_60_). The sandwich device, with the CS–COF–C60 complex (**Figure** **10**) as a functional photoconducting layer, exhibited a fast response to light flashes with a remarkable on/off ratio up to 5.9 × 10^7^ and power efficiency of 0.9%.

## 4 Conclusions and Prospective

P‐FANs represent a new type of porous organic polymers. Compared with other classes of porous organic materials, P‐FANs offer some notable advantages, such as low density, high porosity, tailorable features, functionality, and physicochemical stability. Because of their versatile covalent linkages, they can form various organic building blocks, consisting of only light elements (C, N, H, and O). These advantages, along with the well‐ordered porous structures, endow P‐FAN materials with remarkable potential value in globally important applications, including catalysis, optoelectronics, proton conduction, gas adsorption, storage, and so on. Researchers with diverse areas of expertise are becoming interested in this new field, given the exciting potential. This concise Review covers most of the literature related to P‐FANs and summarizes recent progress in the synthesis, design, and applications of P‐FANs.

To date, the main challenge in the synthesis of P‐FANs is forming highly crystalline structures with robust and stable frameworks. Even though synthesized P‐FANs exhibit a modest PXRD pattern because of partial crystallinity, the overall structural ordering is not yet high enough. Moreover, the majority of the synthesized P‐FANs are 2D‐layered structures. To exploit these materials for gas adsorption and storage, further efforts are needed to construct highly twisted 3D P‐FANs. However, this will not be an easy task because of the difficulty of structural identification, which is a prerequisite for further characterization and applications.

From a synthesis point of view, further advances toward the efficient synthesis of P‐FANs with low‐cost, enhanced crystallinity and mass‐scale production are needed.

The synthesis and application of P‐FANs is a burgeoning new research area. As outlined earlier, there is an enormous scope for the design and synthesis of new P‐FANs with specific combinations of properties. Future collaboration between synthetic chemists and device physicists will permit this research area to flourish.

## Conflict of Interest

The authors declare no conflict of interest.
